# Cellular Immune Responses for Squamous Cell Carcinoma Antigen Recognized by T Cells 3 in Patients with Hepatocellular Carcinoma

**DOI:** 10.1371/journal.pone.0170291

**Published:** 2017-01-23

**Authors:** Kiichiro Kaji, Eishiro Mizukoshi, Tatsuya Yamashita, Kuniaki Arai, Hajime Sunagozaka, Kazumi Fushimi, Hidetoshi Nakagawa, Kazutoshi Yamada, Takeshi Terashima, Masaaki Kitahara, Shuichi Kaneko

**Affiliations:** Department of Gastroenterology, Graduate School of Medicine, Kanazawa University, Kanazawa, Ishikawa, Japan; Fujita Health University, School of Medicine., JAPAN

## Abstract

**Background & Aims:**

Squamous cell carcinoma antigen recognized by T cells 3 (SART3), a tumor-associated antigen expressed in many cancers, functions in tumor rejection. In this study, we investigated its usefulness as an immunotherapeutic target in hepatocellular carcinoma (HCC).

**Methods:**

The expression of SART3 in hepatoma cell lines and HCC tissues was investigated by immunofluorescence and immunohistochemical analyses. Two peptides derived from SART3 (SART3_109_ and SART3_315_) were used for immunological analysis. T-cell responses were investigated by interferon-gamma (IFN-γ) enzyme-linked immunospot and cytotoxic T lymphocyte (CTL) assays using peripheral blood mononuclear cells (PBMCs) in 47 patients, and tumor-infiltrating lymphocytes in 8 of 47 patients with HCC. The safety of immunotherapy using a SART3-derived peptide was investigated by vaccinations of SART3_109_ in 12 patients with HCC (trial registration: UMIN000005677).

**Results:**

The immunofluorescence and immunohistochemical analyses showed that SART3 was expressed in six HCC cell lines, and in HCC tissues including of alpha-fetoprotein-negative individuals. SART3-specific CTLs were generated by stimulating PBMCs with the peptides, and they showed cytotoxicity against HCC cells expressing the protein. Of the 47 HCC patients, 25.5% and 10.6% showed significant responses to SART3_109_ and SART3_315_, respectively. The infiltration of SART3_109_-specific IFN-γ-producing CTLs into the tumor site was confirmed. In the vaccination study, no severe adverse events were observed, and the peptide-specific CTLs were newly induced in four of five patients tested.

**Conclusions:**

SART3 is an immunotherapeutic candidate, and peptides from this antigen may be applied in HCC immunotherapy.

**Trial Registration:**

UMIN000005677

## Introduction

Hepatocellular carcinoma (HCC) is the third most common cause of cancer-related death [1] and its recurrence rate is very high [2]. To protect against recurrence, tumor antigen-specific immunotherapy is an attractive option. During the last two decades, various tumor antigens associated with HCC have been identified as potential candidates for peptide vaccines [3–5]. We have also identified several HCC-specific epitopes, and have used some of them for human trials of HCC immunotherapy [6–9].

Squamous cell carcinoma antigen recognized by T cells 3 (SART3) is a tumor-associated antigen (TAA), and is also known as the human homolog of precursor RNA processing, gene 24 (Prp24) [10]. It is expressed in many malignant tumor cell lines and functions in tumor rejection [11, 12]. In addition, peptides containing SART3 epitopes are capable of generating cytotoxic T lymphocytes (CTLs) and thus have been reported to be effective in immunotherapy to treat several kinds of cancer [13–15]. These reports suggest that SART3 might be useful as a target antigen for HCC immunotherapy.

In this study, we examined SART3 expression in various hepatoma cell lines and HCC tissues of patients, and analyzed immune responses to SART3 using peripheral blood mononuclear cells (PBMCs) and tumor-infiltrating lymphocytes (TILs). Furthermore, to confirm the usefulness of HCC immunotherapy targeting SART3, we analyzed the safety and cellular immune responses induced in patients vaccinated with SART3-derived peptide.

## Patients and Methods

### Patients

**Fig 1:**
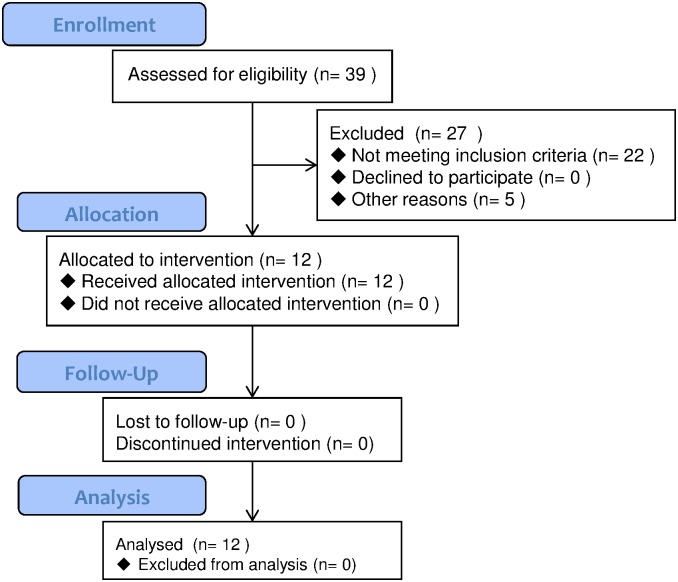
Flow chart of the vaccination study.

Fifty-seven human leukocyte antigen (HLA)-A24-positive HCC patients were examined for cellular immune responses against SART3-derived peptides. In the vaccination study, twelve HLA-A24-positive HCC patients who were treated in Kanazawa University Hospital by radiofrequency ablation (RFA) and achieved complete necrosis of the tumor with a safety margin between June 1, 2011 to March 31, 2013 were enrolled (Fig 1 and S1 and S2 Files). The follow-up period in the vaccination study was March 31, 2015. The diagnosis of HCC was histologically confirmed via ultrasound (US)-guided needle biopsy specimens in 18 cases, surgical resection in 11 cases, and autopsy in 4 cases. For the remaining 14 patients, the diagnosis was based on angiography and/or dynamic computed tomography (CT) [16]. The pathological grading of tumor cell differentiation was assessed according to the general rules for the clinical and pathological study of primary liver cancer [17]. The severity of liver disease was evaluated according to the criteria of Desmet et al. using biopsy specimens of liver tissue [18]. In total, 19 healthy normal donors, 22 chronic hepatitis C patients (including 10 cirrhotic patients) with HLA-A24, who were diagnosed by liver biopsy, and 12 patients with surgical resection of HCC, served as controls. Written informed consent was obtained from each patient. The study protocol conformed to the ethical guidelines of the 1975 Declaration of Helsinki, reflected in *a priori* approval by the regional ethics committee (Medical Ethics Committee of Kanazawa University, No. 1017).

### Cell lines

Six human hepatoma cell lines (HLF, Hep3B, Alex, HepG2, HLE, and Huh7), PaCa-2 (pancreatic cancer cell line), and the HLA-A*2402 gene-transfected C1R cell line (C1R-A24) was cultured as described in detail previously [9].

### Immunofluorescence and immunohistochemical analyses

The expression of SART3 was examined by Immunofluorescence and immunohistochemical analyses described in detail previously [9] with the following modifications. For immunofluorescence analyses, the six different hepatoma cell lines listed above and PaCa-2 (as a positive control) were fixed in acetone with methanol for 5 min and incubated with rabbit anti-human SART3 (Aviva Systems Biology, San Diego, CA, USA; 1:125) or mouse anti-human alpha-fetoprotein (AFP) (Nichirei Bioscience, Tokyo, Japan) antibody overnight at 4°C. Alexa Fluor^®^ 488-conjugated anti-rabbit (Invitrogen, Eugene, OR, USA; 1:500) and anti-mouse immunoglobulin G (IgG) (Invitrogen, Tokyo, Japan) were used for SART3 and AFP detection, respectively. Expression of SART3 and AFP in HCC tissue was examined using both cancerous and non-cancerous tissue obtained from 26 patients. The tissues sections were deparaffinized, treated in a pressure cooker for 1–4 min, and incubated with rabbit anti-human SART3 or AFP (Dako Cytomation, Inc., Carpinteria, CA, USA) antibody overnight at 4°C. The tissue sections were visualized using the Dako EnVision^™^+ System (Dako Cytomation). A semi-quantitative, four-category classification system (negative: no staining, low: < 20% of the area stained, moderate: 20–80% of the area stained, high: > 80% of the area stained) was used to compare expression levels, as described in our previous report [9].

### Enzyme-linked immunospot (ELISpot) assay

The PBMCs and TILs were isolated as described previously [8]. ELISpot assays were performed as reported previously with the following modifications [8, 9]. Two different peptides derived from SART3—SART3_109_ (VYDYNCHVDL) and SART3_315_ (AYIDFEMKI)—were used for the detection of CTLs. They correspond to the HLA-A24-restricted CTL epitope [11, 12]. A human immunodeficiency virus (HIV) envelope-derived peptide (HIVenv_584_) was used as a negative control [19]. 10 ng/mL phorbol 12-myristate 13-acetate (PMA, Sigma) or a cytomegalovirus (CMV) pp65-derived peptide (CMVpp65_328_) was used as a positive control [20]. The peptides were synthesized at Sumitomo Pharmaceuticals (Osaka, Japan) and Genemed Synthesis, Inc. (San Antonio, TX, USA). Responses to peptides were considered positive if the number of specific spots was more than 10, and if the number of spots in the presence of an antigen was at least twofold greater than the number in its absence.

### CTL induction and cytotoxicity assay

SART3_109_ (VYDYNCHVDL) was used to produce SART3-specific T cells. CTLs were expanded from PBMCs as detailed previously [8, 9]. C1R-A24 cells and human hepatoma cell lines were used as targets. Cytotoxicity was evaluated using a chromium-release assay. The percent cytotoxicity was calculated as previously described [8]. For the assay using the hepatoma cell lines, cytotoxicity was considered positive when it was higher than that of CTLs against K562, showing non-specific lysis.

### Vaccination study

To investigate the safety and immunological effects of SART3-derived peptide in HCC patients, twelve HLA-A24-positive HCC patients were enrolled in the present vaccination study. The self-selection method was used in patient recruitment. They were vaccinated with SART3_109_ into the subcutaneous tissue of the armpit from 4 weeks after RFA and were observed for 3months after vaccination. The peptide utilized in the present study was prepared under conditions of Good Manufacturing Practice (NeoMPS, Inc., San Diego, CA, USA). The method of manufacturing a peptide vaccine and vaccination to the patients were similar to our previous report [9]. In detail, 0.04–4 mg/mL peptide solution and incomplete Freund’s adjuvant (Montanide ISA-51; Seppic SA, Paris, France) were mixed and emulsified as a peptide vaccine, and a total of 1.5 mL of the vaccine was injected to the patients received three vaccinations biweekly. Every two weeks, toxicity of the vaccine was assessed using the National Cancer Institute’s Common Terminology Criteria for Adverse Events (ver. 3.0). Of These patients, three patients each were treated with 1/100 (0.03 mg) or 1/10 (0.3 mg) dose of the peptide. After confirming the safety of the peptide, the remaining six patients received 3 mg of the peptide. The study was designed as a dose escalation study and its trial registration number was UMIN000005677.

To investigate the immunological effect, the ELISpot assay using PBMCs obtained before vaccination and at 4 weeks after the final vaccination was performed. Responses to vaccination were considered positive if more than 10 specific spots were detected and if the number of spots after vaccination was at least twofold that before vaccination. After final vaccination, HCC recurrence was evaluated by dynamic CT or magnetic resonance imaging (MRI) every 3 months.

### Statistical analysis

Data are expressed as means ± standard deviation. The Mann-Whitney U test was used for statistical analyses of SART3 expression in HCC and non-cancerous liver tissues. The χ^2^ test with Yates’ correction and the unpaired t-test were used for univariate analysis of the effects of variables on the T-cell response against SART3. A two-sided *P*-value < 0.05 was considered significant.

## Results

### Patient profile

The clinical profiles of the 19 healthy normal donors, 12 patients with chronic hepatitis C, 10 patients with liver cirrhosis, and 47 patients with HCC analyzed in the present study are shown in Table 1. Using the TNM Classification of Malignant Tumours 7^th^ edition, of the Union for International Cancer Control, 11, 21, 3, 3, 1, 1, and 7 patients were classified as having stage I, II, IIIA, IIIB, IIIC, IVA, and IVB tumors, respectively.

**Table 1:** Characteristics of the patients studied.

	Normal donors ^a^	Chronic hepatitis ^a^	Liver cirrhosis ^a^	HCC
No. of patients	19	12	10	47
Age (years) ^b^	35±8	54±11	59±11	66±8
Sex (M/F)	14/5	7/5	4/6	37/10
ALT (IU/L) ^b^	ND	104±119	89±74	65±32
AFP (ng/ml) ^b^	ND	12±4	87±147	1589±7540
Diff. degree of HCC (well/moderate/poor/ND) ^c^	ND	ND	ND	13/17/3/14
Tumor size (large/small) ^d^	ND	ND	ND	38/9
Tumor multiplicity (multiple/solitary)	ND	ND	ND	30/17
Vascular invasion (+/-)	ND	ND	ND	16/31
TNM stage (I/II/IIIA/IIIB/IIIC/IVA/IVB)	ND	ND	ND	11/21/3/3/1/1/7
Liver function (Child-Pugh A/B/C)	ND	12/0/0	6/4/0	30/14/3
Etiology (HBV/HCV/Others) ^e^	ND	0/11/1	1/6/3	8/35/4

^a^ ND: not determined.

^b^ Data are expressed as the mean ± SD.

^c^ Histological degree of HCC; well: well-differentiated, moderate: moderately differentiated, poor: poorly differentiated, ND: not determined.

^d^ Tumor size was divided into either ‘small’ (≦2 cm) or ‘large’ (>2 cm).

^e^ HBV: hepatitis B virus; HCV: hepatitis C virus.

### Expression of SART3 in hepatoma cell lines and HCC tissues

**Fig 2:**
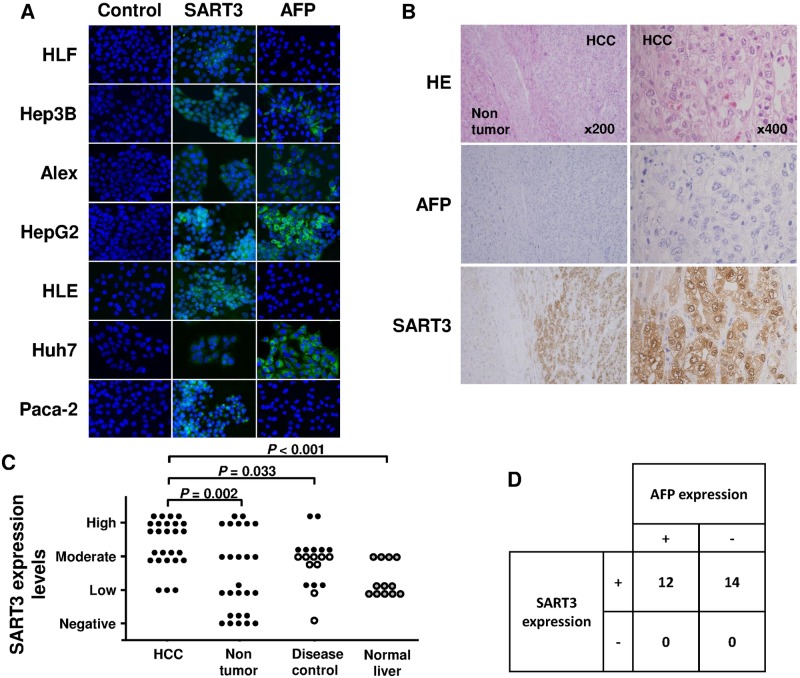
Expression of squamous cell carcinoma antigen recognized by T cells 3 (SART3) in hepatocellular carcinoma (HCC) cell lines and human HCC tissues. (A) Immunofluorescence analysis of the expression of SART3 in hepatoma cell lines. Original magnification, ×400. (B) Immunohistochemical analysis of the expression of SART3 and alpha-fetoprotein (AFP) in sequential non-cancerous and HCC tissue sections. Original magnification, ×200 (left) and ×400 (right). (C) Analysis of SART3 expression levels among the four groups (HCC: tumor tissue in HCC patients, Non-tumor: non-tumor tissue in HCC patients, Disease control: liver tissue in disease control group, Normal liver: tissue from HCC with normal liver patients). Closed and open circles show the level of SART3 expression in the patients with liver cirrhosis and chronic hepatitis, respectively. Grayish circles show the level of SART3 expression in the patients with normal liver. (D) The expression of SART3 was also compared with AFP expression in HCC tissues.

SART3 was expressed in all of the hepatoma cell lines examined. Its distribution was cytoplasmic and nuclear, similar to that for PaCa-2, as reported elsewhere (Fig 2A) [11]. The expression of SART3 was observed even in the hepatoma cell lines not expressing AFP (HLF, HLE).

The expression of SART3 in HCC tissues was examined using samples obtained from 26 HCC patients by US-guided needle tumor biopsy or surgical resection and compared with the expression in non-cancerous tissues. A representative result for one HCC patient is shown in Fig 2B. In this case, SART3 expression was observed in HCC tissue but not in non-cancerous areas, and AFP was not detected in HCC tissue. However, in some cases, the expression of SART3 was observed in non-cancerous tissues.

Therefore, to compare the levels of these proteins between cancerous and non-cancerous tissues, the expression was semi-quantitatively classified into four categories (negative, low, moderate, and high) as described in the Patients and Methods section, and analyzed in 26 patients. The expression of SART3 in non-cancerous tissue was observed in 18 (69%) of 26 patients, and the expression levels of SART3 were higher in HCC tissue than in non-cancerous tissue (*P* = 0.002) (Fig 2C). The expression levels in liver tissue were also observed in disease control group (the patients with chronic hepatitis liver cirrhosis) and normal liver group (the patients with surgical resection of HCC) as controls; however, these expression levels both in disease control group and normal liver group were lower than those in HCC tissue (P = 0.033 and <0.001, respectively). The expression of SART3 and AFP in HCC tissue was observed in 26 (100%) and 12 (46%) of 26 patients, respectively (Fig 2D). The expression of SART3 in the absence of AFP expression was observed in 14 patients.

### Detection of SART3-specific T cells assessed by interferon (IFN)-γ ELISpot analysis

**Fig 3:**
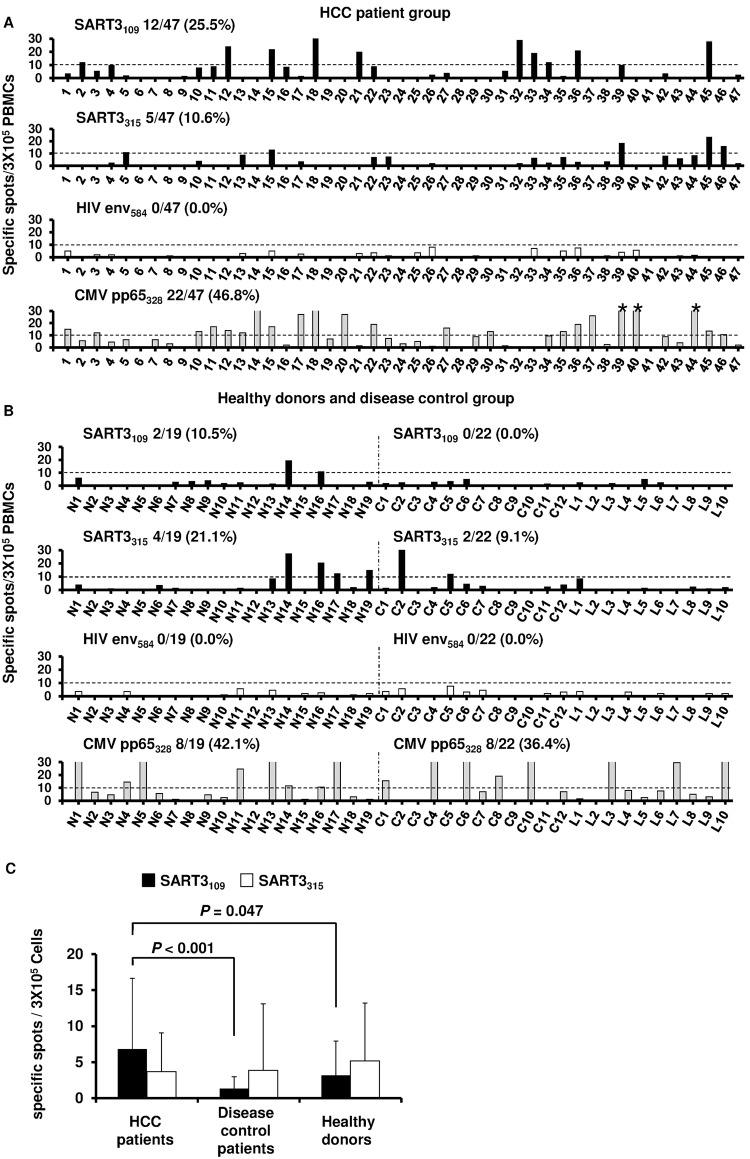
Immune responses of SART3-specific T cells in HCC patients. (A) Interferon (IFN)-γ enzyme-linked immunospot (ELISpot) assay was performed using SART3, HIVenv_584_, and cytomegalovirus pp65_328_-derived peptides with peripheral blood mononuclear cells (PBMCs) of HCC patients. % indicates the proportion of patients who showed positive responses. (B) IFN-γ ELISpot assay in healthy donors and disease control groups. “N” denotes normal donors. “C” denotes the patients with chronic hepatitis. “L” denotes the patients with liver cirrhosis. % shows the proportion of patients who exhibited positive responses. (C) Comparison of the mean number of SART3-derived peptide-specific spots among the three groups. Error bars indicate the standard deviations. Black and white bars indicate the frequencies of SART3_109_- and SART3_315_-specific T cells, respectively.

ELISpot assays were performed using PBMCs from the HCC patients to investigate the frequency of CTLs specific for the peptides derived from SART3 (Fig 3). Of the 47 HCC patients, 25.5% (12 patients) and 10.6% (5 patients) showed significant responses to SART3_109_ and SART3_315_, respectively (Fig 3A). The same analyses were performed in the healthy donors and disease control group (patients with chronic hepatitis and cirrhosis without HCC). Among the 19 healthy donors, 10.5% (2 donors) and 21.1% (4 donors) showed significant responses to SART3_109_ and SART3_315_, respectively. Among the 22 patients of the disease control group, 0.0% and 9.1% (2 patients) showed significant responses to SART3_109_ and SART3_315_, respectively (Fig 3B). Significant differences in the frequency of SART3_109_-specific spots were detected between patients with HCC, disease-control patients, and healthy donors (Fig 3C). On the other hand, no significant differences were detected in those of the SART3_315_ among the three groups. These results suggested that SART3_109_ peptide was preferable as a peptide vaccine. Significant responses specific to CMVpp65_328_ were detected in 46.8%, 42.1%, and 36.4% of HCC patients, healthy donors, and the disease control group, respectively, with no significant difference among these three groups. No patients showed a significant response specific to HIVenv_584_.

To clarify the clinical characteristics of SART3-specific T-cell responses in HCC patients, the clinical background was compared between patients who showed positive responses to SART3-derived peptides in the ELISpot assay and those who did not. The clinical features of both groups were not significantly different in terms of age, gender, serum AFP levels, differentiation of HCC, tumor multiplicity, vascular invasion, TNM factors and stages, histology of the non-tumor liver, liver function, or the type of viral infection (S1 Table). SART3-specific T cells could be generated even in the early stages of HCC and independent of a hepatitis viral infection.

### Detection of SART3-specific T cells in TILs

**Fig 4:**
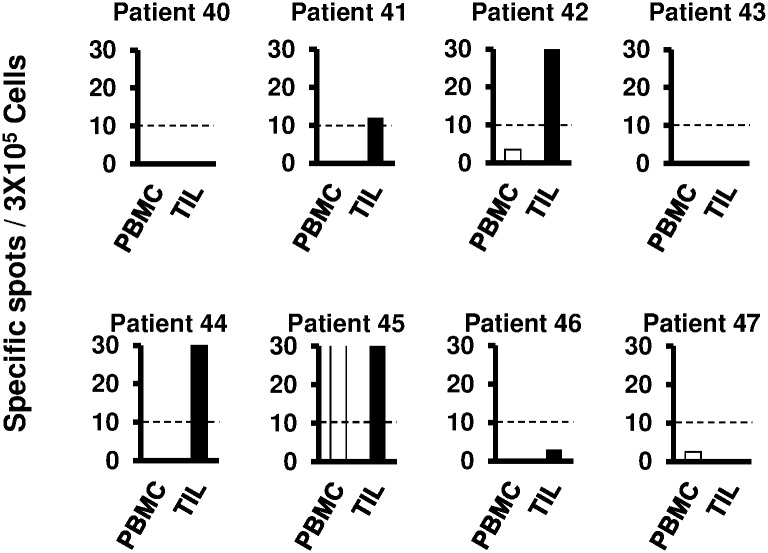
IFN-γ ELISpot assay using tumor-infiltrating lymphocytes (TILs) against one of the SART3-derived peptides (SART3_109_) in HCC patients. ELISpot was performed in eight patients from whom samples of both PBMCs and TILs could be obtained. Open and solid bars show the frequency of SART3-specific T cells in PBMCs and TILs, respectively.

Next, to examine the existence of SART3-specific T cells in TILs, we performed a similar analysis in another eight patients from whom samples of both PBMCs and TILs could be obtained. In the assay using PBMCs and TILs, one (12.5%) and four (50.0%) of the eight patients, respectively, showed significant responses to SART3_109_ (Fig 4). SART3-specific T cells in TILs were observed even in patients without SART3-specific T-cell responses in PBMCs.

### Comparison of SART3-specific T-cell responses with AFP-specific T-cell responses

To compare SART3-specific and AFP-specific T-cell responses, an IFN-γ ELISpot assay using an AFP-derived peptide (AFP_403_) [6] was also performed in the same HCC patients(S1 Fig). A significant response was detected in 10.6% (S1A Fig) of these patients. In a comparison between the mean numbers of specific spots for SART3_109_ and for AFP_403_, the frequency of SART3_109_-specific T cells was not higher than that of AFP_403_ in all patients (S1B Fig). However, in the patients who showed significant responses for either SART3_109_ or AFP_403_, the frequency of SART3_109_-specific T cells was significantly higher than that of AFP_403_ (S1C Fig).

### Cytotoxic activity of SART3-specific T cells against hepatoma cell lines

**Fig 5:**
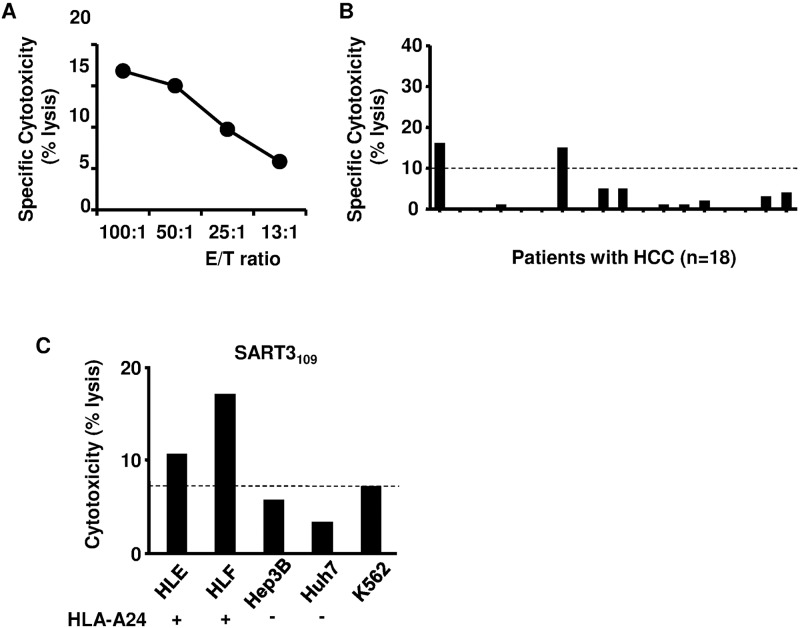
Induction of SART3-specific T cells in HCC patients. (A) Representative results of the cytotoxic T lymphocyte (CTL) assay. The results are shown as specific cytotoxic activity, which was calculated as follows: (cytotoxic activity in the presence of peptide)–(cytotoxic activity in the absence of peptide); findings were considered positive at > 10%. (B) CTL assays [effector-to-target (E/T) ratio of 50:1] were performed in 18 HCC patients. The results are also shown as specific cytotoxic activity. Solid bars show the results for one patient. (C) Cytotoxicity of SART3-specific T-cell lines derived from the peptide was also measured against hepatoma cell lines. The cytotoxicity was considered positive when it was higher than that against K562, which shows non-specific lysis (E/T ratio of 50:1). Representative results are shown.

We investigated whether SART3_109_ is capable of generating peptide-specific CTLs from PBMCs. Because of the limitation of blood samples, CTL assay using PBMCs from 47 HCC patients could not be performed. Thus, PBMCs derived from other 18 HCC patients were used to assess the cytotoxicity (Fig 5 and S2 Table). The induction of SART3-specific T cells by peptide stimulation was observed in 2 of 18 patients (Fig 5A). These CTLs also exhibited cytotoxicity against hepatoma cell lines with the HLA-A24 molecule and expression of SART3, which correspond to HLE and HLF, but not against Hep3B and Huh7 cells without HLA-A24 (Fig 5B).

### Clinical safety of SART3_109_ peptide and its immunological effects

**Fig 6:**
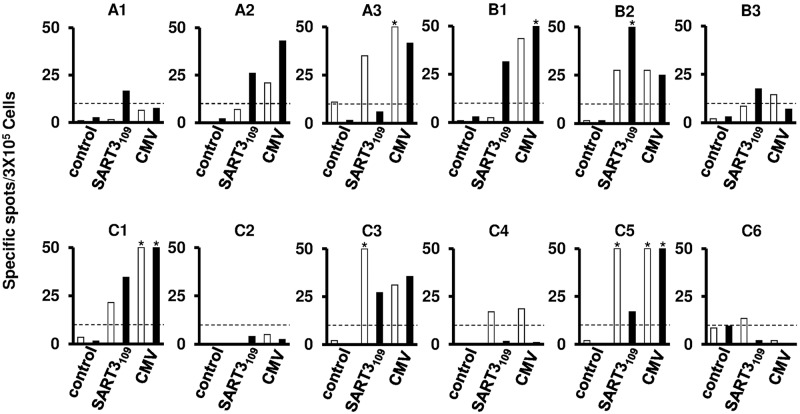
Interferon(IFN)-γ enzyme-linked immunospot assays of PBMCs to SART3-derived peptide (SART3_109_) or control peptides (CMVpp65_328_) in HCC patients with RFA and SART3_109_ peptide vaccination. White and black bars show the T-cell responses before and after vaccination, respectively. An asterisk denotes more than 50 specific spots.

The clinical profiles of the 12 HCC patients with vaccination are shown in Table 2. The patients in group A, B and C were vaccinated with 0.03, 0.3 and 3 mg of the peptide, respectively. The treatment was well-tolerated and there were no serious adverse events. The most common adverse event was grade 1 injection-site reaction manifesting as pain, pruritus, and skin induration. Worsening of hepatitis or liver function was not observed in any of the vaccinated patients. Positive T-cell responses after vaccination were observed in 8 of 12 (66.7%) patients: two of three (66.7%) patients in group A, three of three (100.0%) in group B, and three of six (50.0%) patients in group C. In four of five (80.0%) patients without positive T-cell responses before vaccination, the SART3-derived peptide-specific CTLs were newly induced (Fig 6). The median relapse-free survival of group A, B, and C were 280, 329 and 536 days, respectively (S2 Fig). Although there was no statistical significance on account of the small number of patients, the recurrence rate in the patients with the CTL responses after vaccination (3 of 8 patients, 37.5%) was lower than that in the patients without CTL responses (2 of 4 patients, 50%) at 300 days after RFA (Table 2).

**Table 2:** Patient characteristics of vaccination study.

Patient	Peptide Dose (mg)	Age (years)	Sex	Etiology ^a^	Stage of HCC	ALT (IU/L)	AFP (ng/ml)	Child-Pugh (A/B/C)	Histology of liver ^b^	Treatment ^c^	Immune Response (before/after)	Toxicity ^d^ (grade)	Recurrence (at 300 days after vaccination)	Relapse-free Survival (days)
A1	0.03	69	M	HBV	II	26	5	A	ND (LC)	RFA	-/+	None	+	280
A2	0.03	71	M	Others	III	24	4	A	F3	TACE, RFA	-/+	P(1), S(1)	+	113
A3	0.03	67	M	HCV	II	34	4	A	F3A1	RFA	+/-	Pa(1)	-	442
B1	0.3	62	M	HCV	I	78	49	A	F3A2	RFA	-/+	None	-	329
B2	0.3	66	M	HCV	II	37	11	A	F4A2	RFA	+/+	None	+	189
B3	0.3	74	M	HBV	II	50	2	A	F3A2	RFA	-/+	P(1), S(1)	-	460
C1	3	67	F	HCV	II	35	11	A	F2A1	RFA	+/+	P(1), S(1)	-	536
C2	3	69	F	Others	II	22	24	A	ND (LC)	RFA	-/-	P(1), S(1)	+	133
C3	3	69	M	HCV	I	27	8	A	F4A1	RFA	+/+	Pa(1)	-	810
C4	3	72	M	HCV	I	31	8	A	F3A2	RFA	+/-	P(1), S(1)	+	260
C5	3	77	F	HBV	II	22	183	A	F4A1	RFA	+/+	P(1), S(1)	-	647
C6	3	83	M	HCV	I	24	4	A	F1A1	RFA	+/-	None	-	693

^a^ HBV: hepatitis B virus; HCV: hepatitis C virus.

^b^ Histology of the liver; ND: not determined, LC: liver cirrhosis.

^c^ Treatment of HCC; RFA: radiofrequency ablation, TACE: transarterial chemoembolization

^d^ Toxicity; P: Pruritus, Pa: Pain, S: Skin induration.

## Discussion

Several TAAs and their CTL epitopes expressed in HCC have been discovered over the past 10–15 years [6–9, 21–24] and several trials of TAA-derived peptides for the treatment of HCC (e.g., AFP, human telomerase reverse transcriptase, and glypican-3) have been performed [25–27]. In these studies, the anti-tumor effects were very limited. Therefore, there is still a need to identify other TAAs as therapeutic targets for HCC immunotherapy.

The important factors to consider when determining the usefulness of TAAs for peptide vaccine therapy are the immunogenicity, specificity, oncogenicity, expression levels, percentage of positive cells, and number of patients with antigen-positive cancer [28]. In the present study, the expression of SART3 was confirmed in 6/6 (100%) hepatoma cell lines. Furthermore, the expression of SART3 was detected in the HCC tissue of all patients, and was independent of the expression of AFP in the tumor. These results suggest the advantage of this antigen as a target of immunotherapy for HCC. However, the expression of SART3 was also observed in non-cancerous tissues of HCC patients and liver tissue in the patients with chronic hepatitis or liver cirrhosis without HCC, although less frequently and at lower levels than in HCC tissue. Our results are consistent with the recent findings that SART3 is actually the human homologue of Prp24p, and is expressed in some normal tissues including liver tissue [10]. Such results imply that immunotherapy targeting SART3 may have adverse effects on liver tissue expressing the protein. However, previous studies using SART3-derived peptides as a vaccine for several cancers have found them to be safe, suggesting that immunotherapy is applicable to HCC [15, 29]. The results of the clinical trial that we performed as part of the present study also showed the safety of SART3-derived peptides, even in the patients with chronic hepatitis or cirrhosis.

With regard to the immunogenicity of SART3, we showed that SART3-specific CTLs could be generated by stimulating PBMCs with peptides, and the CTLs were cytotoxic to hepatoma cell lines. In the ELISpot assay, SART3-specific immune responses were observed frequently only in HCC patients, and the frequency of CTLs was higher in the HCC patients than in the control groups. CTLs were also detected among TILs, suggesting that they infiltrate into the tumor. In a vaccination study with a SART3-derived peptide, seven patients had a SART3-derived peptide-specific T-cell response before vaccination, and newly induced SART3-specific CTLs were observed in four of five (80.0%) patients; this is similar to the frequency of responsive patients reported in other peptide vaccination studies [26, 27], suggesting that SART3_109_ is an immunogenic peptide. On the other hand, SART3-specific immune responses were lower in patients C3 and C5 and had disappeared in patients A3, C4, and C6, suggesting possible changes in circulating CTL precursors. The CTLs activated by vaccination might have infiltrated the tumor tissues and induced an immune response to the tumor cells. Thus, it became difficult to detect CTLs in the PBMCs of the patients. Our ELISpot data using PBMCs and TILs (Fig 4) might support this speculation.

Apart from the induction of CTLs, the efficacy of SART3-derived peptides for a vaccine for advanced HCC is still unclear. Other recent studies using SART3-derived peptides for other advanced cancers showed the induction of cellular immune responses and clinical responses for certain patients [15, 29]. Upon analyzing the prognosis of patients with RFA and SART3-derived peptide vaccination in the present study, the recurrence rate in the patients with an increase in the peptide-specific CTLs after vaccination was lower than that in the patients without an immune response. Although further studies are necessary to evaluate the efficacy of SART3-derived peptides for HCC, taken together with the results of previous studies, our results suggest that SART3 can be considered an optimal antigen that satisfies most of the criteria [28].

In conclusion, SART3 is a potential candidate for a TAA with immunogenicity, and peptides derived from this protein may be applied to HCC immunotherapy.

## Supporting Information

S1 Fig Comparison of the frequency of squamous cell carcinoma antigen recognized by T cells 3 (SART3) and alpha-fetoprotein (AFP)-specific T cells. (A) Interferon-γ enzyme-linked immunospot assays were performed using peripheral blood mononuclear cells of hepatocellular carcinoma patients against AFP-derived peptides. (B) Comparison of the frequency of AFP_403_ and SART3_109_-specific T cells in all patients. (C) Comparison of the frequency of AFP_403_ and SART3_109_-specific T cells in patients who showed a positive T-cell response.
(TIF)

S2 Fig Relapse-free intervals of the vaccinated patients. The median relapse-free intervals were 280, 329, and 536 days in groups A, B, and C, respectively, although the peptide-specific responses in group C tended to be lower.
(TIF)

S1 File Protocol for a clinical trial in English. (DOC)

S2 File Protocol for a clinical trial in original language (in Japanese). (DOC)

S1 Table Univariate analysis of the effects of variables on the T cell response against SART3. (TIF)

S2 Table Characteristics of the patients evaluated by the cytotoxicity assay. (TIF)

S1 TREND Checklist (PDF)
