# Supplementary figures and images for: Cellular Immune Responses for Squamous Cell Carcinoma Antigen Recognized by T Cells 3 in Patients with Hepatocellular Carcinoma
Source: PLoS One. 2017 Jan 23;12(1):e0170291. doi: 10.1371/journal.pone.0170291 (PMC5256867; doi:10.1371/journal.pone.0170291)

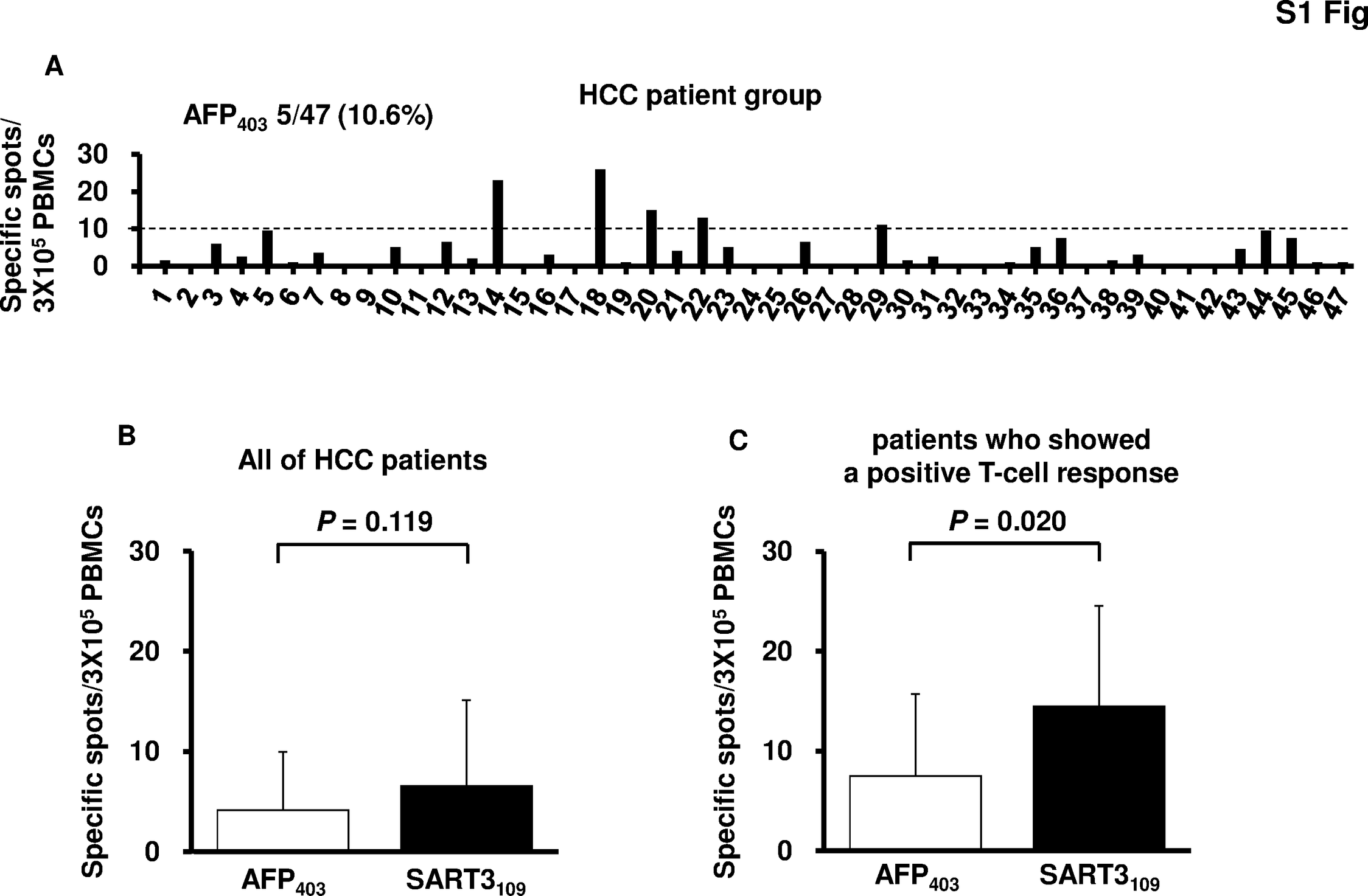

Supplement: S1 Fig — (A) Interferon-γ enzyme-linked immunospot assays were performed using peripheral blood mononuclear cells of hepatocellular carcinoma patients against AFP-derived peptides. (B) Comparison of the frequency of AFP403 and SART3109-specific T cells in all patients. (C) Comparison of the frequency of AFP403 and SART3109-specific T cells in patients who showed a positive T-cell response. (TIF) [file pone.0170291.s001.tif]

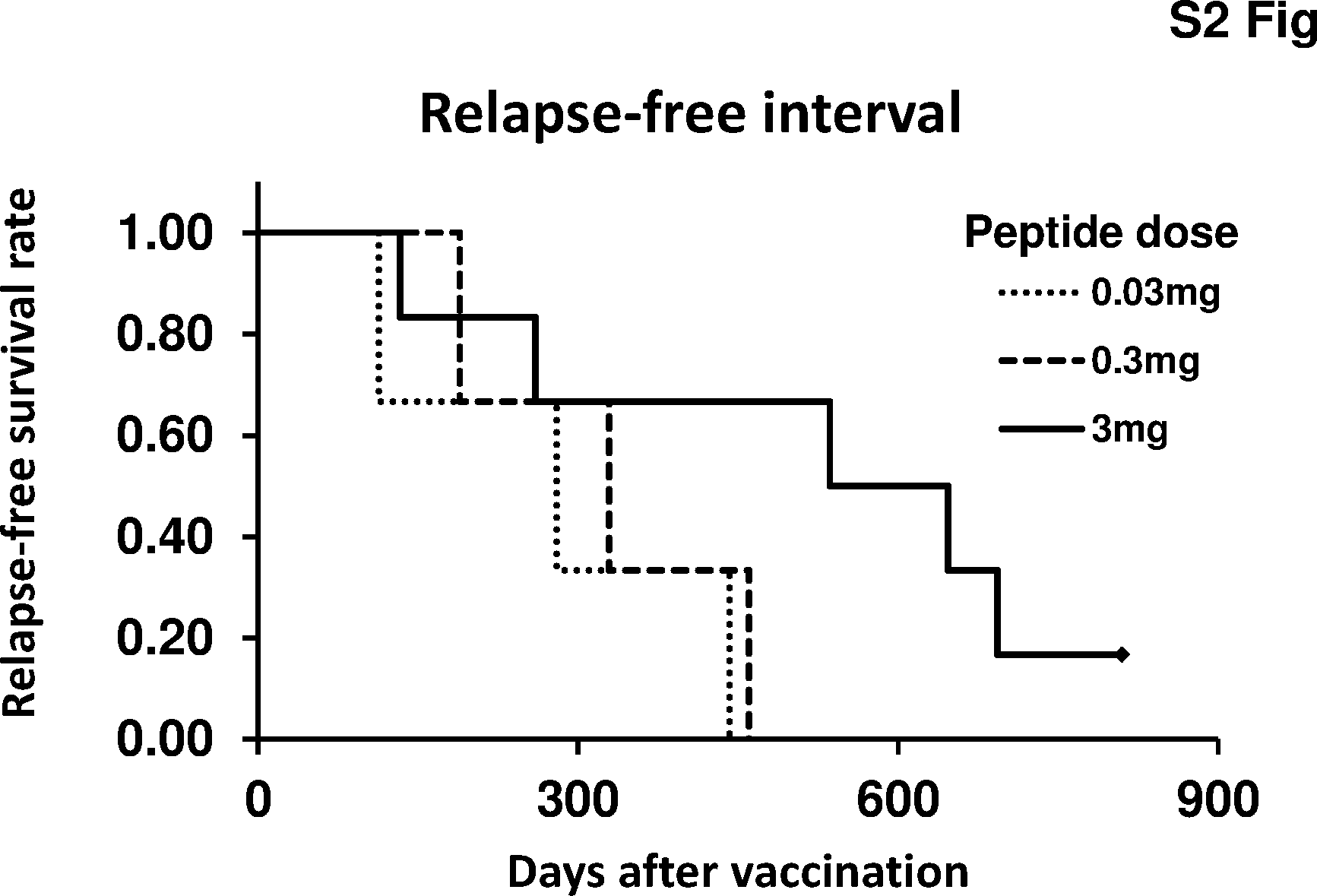

Supplement: S2 Fig — The median relapse-free intervals were 280, 329, and 536 days in groups A, B, and C, respectively, although the peptide-specific responses in group C tended to be lower. (TIF) [file pone.0170291.s002.tif]

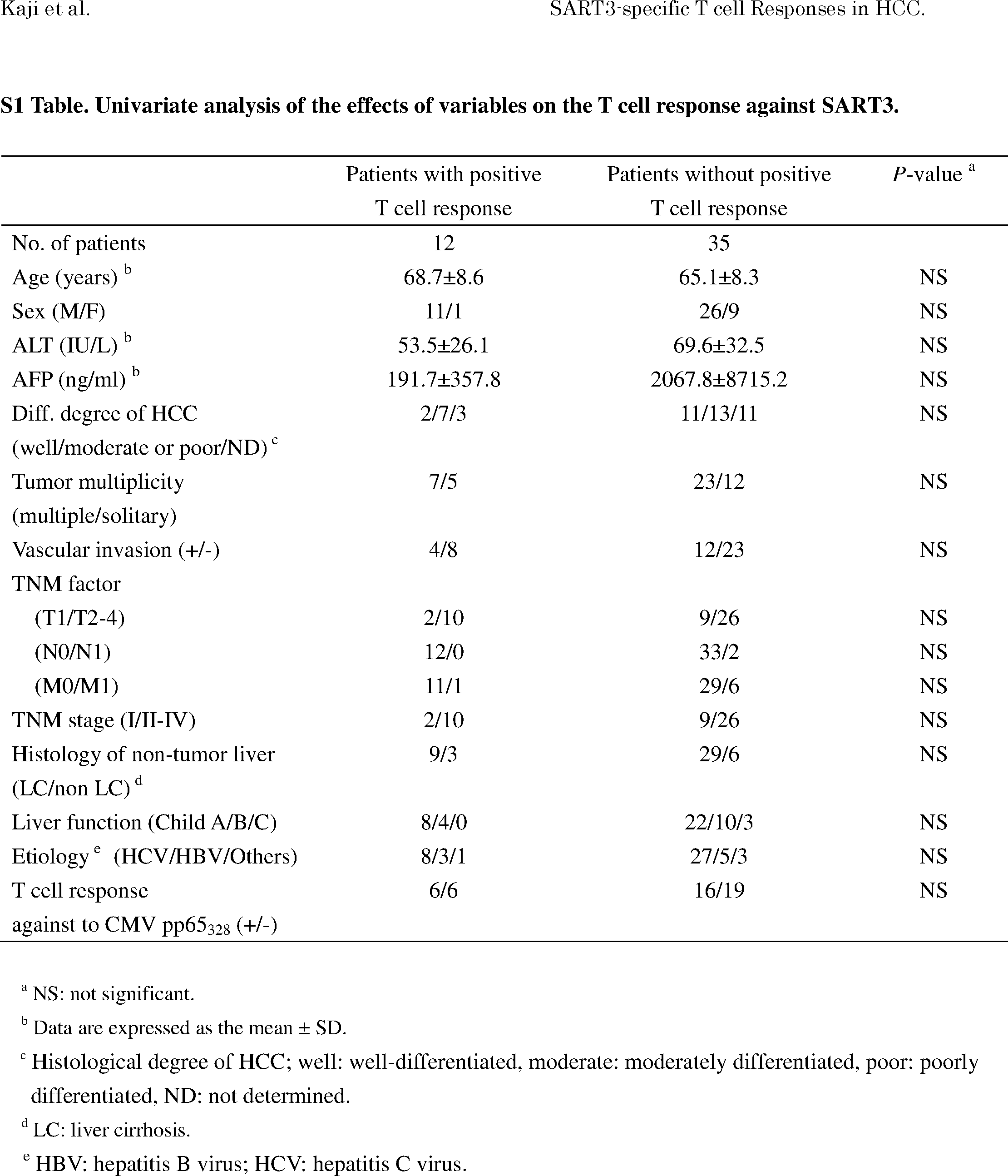

Supplement: S1 Table — (TIF) [file pone.0170291.s005.tif]

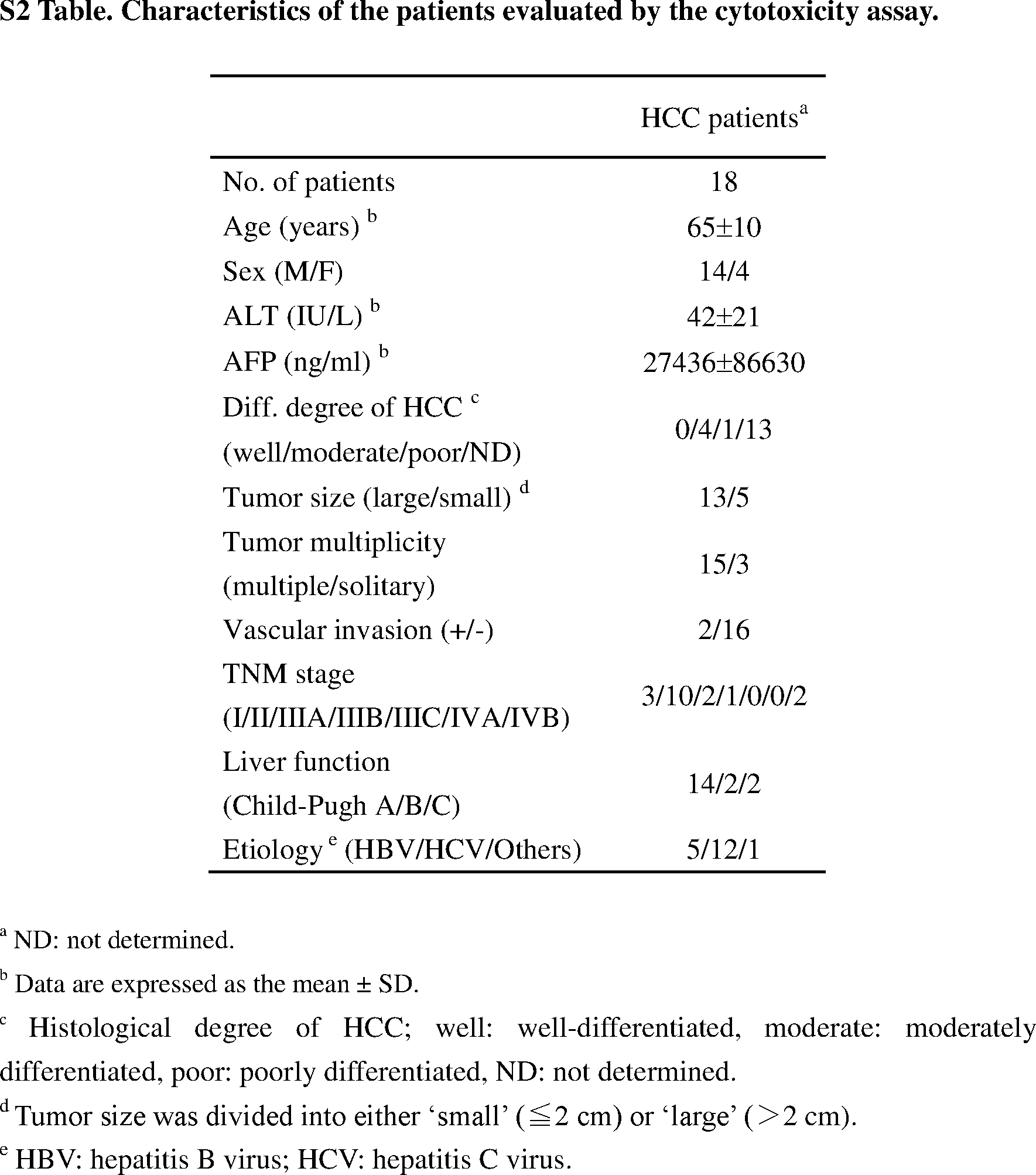

Supplement: S2 Table — (TIF) [file pone.0170291.s006.tif]
