# Supplementary material for: Cellular Immune Responses for Squamous Cell Carcinoma Antigen Recognized by T Cells 3 in Patients with Hepatocellular Carcinoma
Source: PLoS One. 2017 Jan 23;12(1):e0170291. doi: 10.1371/journal.pone.0170291 (PMC5256867; doi:10.1371/journal.pone.0170291)
Supplement: S1 File — (DOC) [file pone.0170291.s003.doc]

Protocol for a clinical trial on “Immunotherapy for hepatocellular cancer using Prp24p human homologue RNA binding nucleoprotein-derived peptide vaccine”

To Chairman of the Kanazawa University Medical Ethics Review Board

**1. Request**

We request the board to review the antitumor effects of immunotherapy for hepatocellular cancer using Prp24p human homologue RNA binding nucleoprotein (SART3)-derived peptide vaccine in patients with hepatocellular carcinomas incurable by surgery.

**2. Principles of peptide vaccine treatment**

Since the identification of the melanoma antigen *MAGE* gene by Boon et al. in 1991, the human immune system has been demonstrated to recognize and eliminate cancer. In the process of cancer elimination, T cells play a major role. Specifically, they recognize the complexes of the major histocompatibility antigens (HLA molecules) present on cancer cells and the peptide fragments of proteins produced by the cancer cells to exert cytotoxic activity.

Many cancer rejection antigens and peptides with the amino acid sequences of the antigens have been identified and subjected to clinical trials for cancer immunotherapy throughout the world. These peptides can induce T cells that attack cancer cells *in vivo* and, in some cases, cancer regression (Reference 1).

**3. Objectives**

The annual number of deaths due to hepatocellular carcinoma (liver cancer) in Japan exceeded 30,000 and is further increasing in spite of the implementation of various local treatments, such as surgery, hepatic artery tumor embolization (TAE), and radiofrequency ablation (RFA), and chemotherapy with anticancer agents. Of these, surgery has the most potent therapeutic effects, although its indications are limited to those in very early stages. Thus, treatments other than surgery are selected in most cases of liver cancer. TAE and RFA have been employed as treatments for non-hepatocellular carcinomas incurable by surgery. However, our therapeutic results showed extremely high recurrence rates: 88.4 and 75.2% at three years after TAE and RFA treatments, respectively. Currently, no treatment is available to extend the duration of survival.

To develop a new treatment for liver cancer, we conducted a clinical trial “immunotherapy for hepatocellular carcinoma using dendritic cells” in patients with hepatocellular carcinomas incurable by surgery between October 1, 2001 and March 31, 2003 (acceptance number 236 of the Kanazawa University Medical Ethics Review Board). In this trial, immature dendritic cells were induced from the peripheral blood before TAE treatment and administered through a catheter locally to the tumors of 10 liver cancer patients. As a result, immature dendritic cells could be safely administered. Subsequently, clinical trials “immunotherapy for hepatocellular carcinomas using SART-3; 109-118 peptide-stimulated dendritic cells,” “immunotherapy for hepatocellular carcinomas using OK-432-stimulated dendritic cells,” and “immunotherapy for hepatocellular carcinomas by boost administration of peptide-stimulated dendritic cells” were implemented, demonstrating that:

1) OK-432-stimulated dendritic cells could be safely administered;

2) Dendritic cells could induce liver cancer-specific tumor antigens and immune response against the CTL epitopes more effectively than TAE alone;

3) OK-432-stimulated dendritic cells could induce antitumor immunity and more significantly suppressed recurrence than TAE treatment alone; and

4) Dendritic cells, stimulated with and bound to a peptide, could be safely administered to humans.

These results suggest that recurrence after local treatment may be prevented by inducing antitumor immunity against liver cancer. The peptide (SART3) to be used in the present clinical trial has already been examined in clinical trials at many institutions, and was demonstrated to be safely administered to humans (References 2 and 3). The conventional method with dendritic cells cannot be widely applied to patients, because it requires peripheral blood sampling (200 cc), takes one week to start treatment after the sampling, and demands a special culture environment because of a risk of bacterial contamination due to *in vitro* culturing of dendritic cells after the sampling. To overcome these difficulties and facilitate the treatment in a larger number of patients, a peptide with a liver cancer-specific antigen epitope was subcutaneously administered to induce a liver cancer-specific immune response, with an aim to develop a method to suppress recurrence after local treatment. Hence, the present clinical trial was conducted to examine the safety of administering the peptide to liver cancer patients and the efficiency of the method to induce an antitumor immune response.

The peptide (Prp24p human homologue RNA-binding nuclear protein-derived peptide; SART-3; MPS-88) to be used in the clinical trial is derived from a tumor antigen identified in liver cancer patients by basic research. It is frequently expressed in liver cancer, contains an HLA-A24-restricted CTL epitope which is frequently detected in the lymphocytes of liver cancer patients, and induces CTL *in vitro* (Reference 4). In addition, the peptide to be administered to patients was manufactured as a clinical (GMP) grade product (MPS-88) (NeoMPS® with a GMP certificate) under the quality management by U.S. Neo MPS, Inc. (Appendix 1).

The method to administer the peptide has already been employed in many institutions. Its mixture with an adjuvant Montanide ISA-51, whose safety has been demonstrated, is subcutaneously administered. In our department, basic research is conducted on the safety of the peptide in mice by administering 100 times the amount of peptide per body weight, compared with that administered in the present trial, to demonstrate no change in body weights, activities, and administration site (skin) (Appendix 2).

Primary endpoints

1) Incidence of adverse events due to HLA-A24-restricted SART3-derived peptide vaccine (SART3-109); and

2) Immunological monitoring of specific immune response induction

Secondary endpoints

1) Recurrence rates after local treatment; and

2) Tumor markers after the completion of treatment

**4. Subjects**

Among hepatocellular carcinoma patients who are not indicated for or do not desire surgery, HLA-A24-positive patients to be treated with TAE ^Note 1)^ or RFA ^Note 2)^ were included. Specifically, among those of Stage II or above according to the General Rules for the Clinical and Pathological Study of Primary Liver Cancer (4th edition, November 2001), who are not indicated for surgery, and those of Stage I, for whom surgery cannot be performed because of the lowered liver functional reserve or who do not desire surgery, 12 patients who met the following three criteria were included. Of these, three patients each were treated with 1/100 (0.03 mg) or 1/10 (0.3 mg) dose of the peptide. After confirming the safety of the peptide, the remaining six patients received 3 mg of the peptide.

Note 1) Hepatic artery embolization: The blood vessels are embolized to block the flow of nutrition and oxygen to hepatocellular carcinomas.

Note 2) Radiofrequency ablation: Hepatocellular carcinomas are heat-treated with a needle electrode.

Inclusion criteria

⦁ Definitive diagnosis of primary hepatocellular carcinoma based on imaging findings

⦁ 70% Karnofsky Performance Status

⦁ 20 years or older and informed consent

⦁ 2,000/mm^3^ leukocyte count

⦁ 50,000/mm^3^ platelet count

⦁ 8.5 g/dL hemoglobin level

⦁ Liver damage A or B according to the General Rules for the Clinical and Pathological Study of Primary Liver Cancer (4th edition)

⦁ 1.5 mg/dL serum creatinine level

Exclusion criteria: Patients who met any one of the following criteria were excluded:

⦁ Heart, kidney, respiratory, and blood diseases, coagulation disorders, and other serious complications, diagnosed by attending physicians;

⦁ HIV infection;

⦁ Surgery, chemotherapy, or radiation therapy within four weeks or insufficient recovery from these treatments;

⦁ Diagnosis of immunodeficiency;

⦁ Need of corticosteroid treatment;

⦁ Breastfeeding;

⦁ Pregnancy or possible pregnancy;

⦁ History of allogeneic organ transplantation;

⦁ Difficulties with follow-up observation; and

⦁ Uncertainty regarding protocol compliance.

Registration process

Selection of cases: Attending physicians

Confirmation of cases: Investigator

Explanation about informed consent: Attending physicians

Creation of informed consent: Patients and attending physicians

Drug administration during the treatment

In this clinical trial, the following medications are prohibited:

⦁ Systemic administration of corticosteroids used for conditions other than acute non-allergic reactions; and

⦁ Immunosuppressive agents

**5. Methods**

1) The peptide (SART3-109) (MPS-88) is administered within six weeks after the local treatment of hepatocellular carcinoma, and is subsequently administered a total of three times every two weeks. Before the start of the administration, the typing of HLA class I is conducted by a blood test.

2) Specifically, 4 mg of SART3-derived peptide (SART3-109) (GMP grade) was dissolved in 1 mL of saline, and mixed with 1 mL of Montanide ISA-51 (GMP grade) using two 5-mL injection syringes and a 3-way stopcock, followed by emulsification. The emulsion (0.5 mL each) was dispensed into a tuberculin syringe, and a total of 1.5 ml (subcutaneous injection of 0.25 mL each in a total of six times) was injected subcutaneously (under both armpits). The peptide is diluted at 1/100 (0.03 mg) or 1/10 (0.3mg) with 1 mL of saline before administration.

3) Before peptide administration and at 4 weeks after the final peptide administration, 50 mL each of peripheral blood was collected to examine the induction of peptide-specific immune response using ELISPOT and tetramer assays. This amount is required for lymphocyte isolation to investigate biological reactions to peptide vaccines using the above assays and lymphocyte storage to conduct pathological analyses of unexpected adverse effects.

In addition, delayed type hypersensitivity (DTH) was examined with 10 μg of peptide dissolved in saline. For this purpose, 0.1 mL of peptide lysate was intradermally injected into the forearm, and >4-mm induration at 48 hours, without induration at the reaction site of saline as a control, was determined to be positive for DTH.

4) Therapeutic effects were examined based on recurrence rates at local and other sites, determined by diagnostic imaging at 3, 6, 9, and 12 months after local liver cancer therapy.

5) To examine antitumor immune responses and pathologically analyze unexpected adverse effects, 10 mL each of peripheral blood was collected before and at four weeks after peptide administration, followed by separation and cryopreservation of sera.

6) To examine antitumor immune response, clinical specimens were sent to the Department of Immunology, Toyama University School of Medicine, with an aim to investigate T-cell receptors.

Expected adverse effects

⦁ Fever

⦁ Autoimmune disease

⦁ Exacerbation of hepatitis, lowered liver functional reserve, and liver failure

⦁ Bacterial infection

⦁ Skin reaction at the site of administration; itching, pain, redness, induration, erosion, ulcers, bruises

⦁ Lymph node swelling

⦁ Leukocytosis

⦁ Rash

⦁ Allergy

⦁ Shock

Toxicity

Toxicity and adverse events are determined using the NCI Common Toxicity Criteria (CTC) Scale.

Criteria for discontinuation

⦁ Subjects have a right to voluntarily withdraw from the trial anytime for any reason without affecting subsequent medical treatment according to the Declaration of Helsinki, ICH-GCP guidelines, and US FDA regulations.

⦁ For serious adverse effects, appropriate treatment should be immediately conducted.

Cost of treatment

All costs of treatments, excluding routine hepatocellular cancer treatments covered by health insurance, will be paid by the educational and delegated accounting funds of the Department of Disease Control and Homeostasis, Kanazawa University Graduate School of Medical Sciences.

Compensation for health damage

No compensation is made for any health damage. Sufficient care is exercised for the development of adverse effects. However, if health damage, such as disorders and diseases, occur in this clinical trial, their causes are investigated to take appropriate measures.

Data management

Concatenated patient data are entered after anonymization. The director of the clinical trial should strictly manage the data and store documents in a locked safe. Electronic data should be managed in a password-protected personal computer. The data obtained in the trial should not be used for improper purposes.

**6. Period of the clinical trial**

The registration period of the clinical trial is from the date of approval by the Ethics Committee to March 31, 2013. Adverse events are followed up for three months after the end of peptide administration. The entire clinical trial, including analytical research, is scheduled to end by March 31, 2015.

**7. Contact**

Clinical study director: Shuichi Kaneko, Professor of the School of Medicine, College of Medical, Pharmaceutical and Health Sciences, Kanazawa University

Attending physician of the clinical trial: Eishiro Mizukoshi, Lecturer of the Department of Gastroenterology, Kanazawa University Hospital

13-1, Takara-cho, Kanazawa City, Ishikawa Prefecture, 920-8641

Tel.: 076-265-2235

Fax: 076-234-4250

E-mail: [eishirom@m-kanazawa.jp](mailto:eishirom@m-kanazawa.jp)

Appendix 1


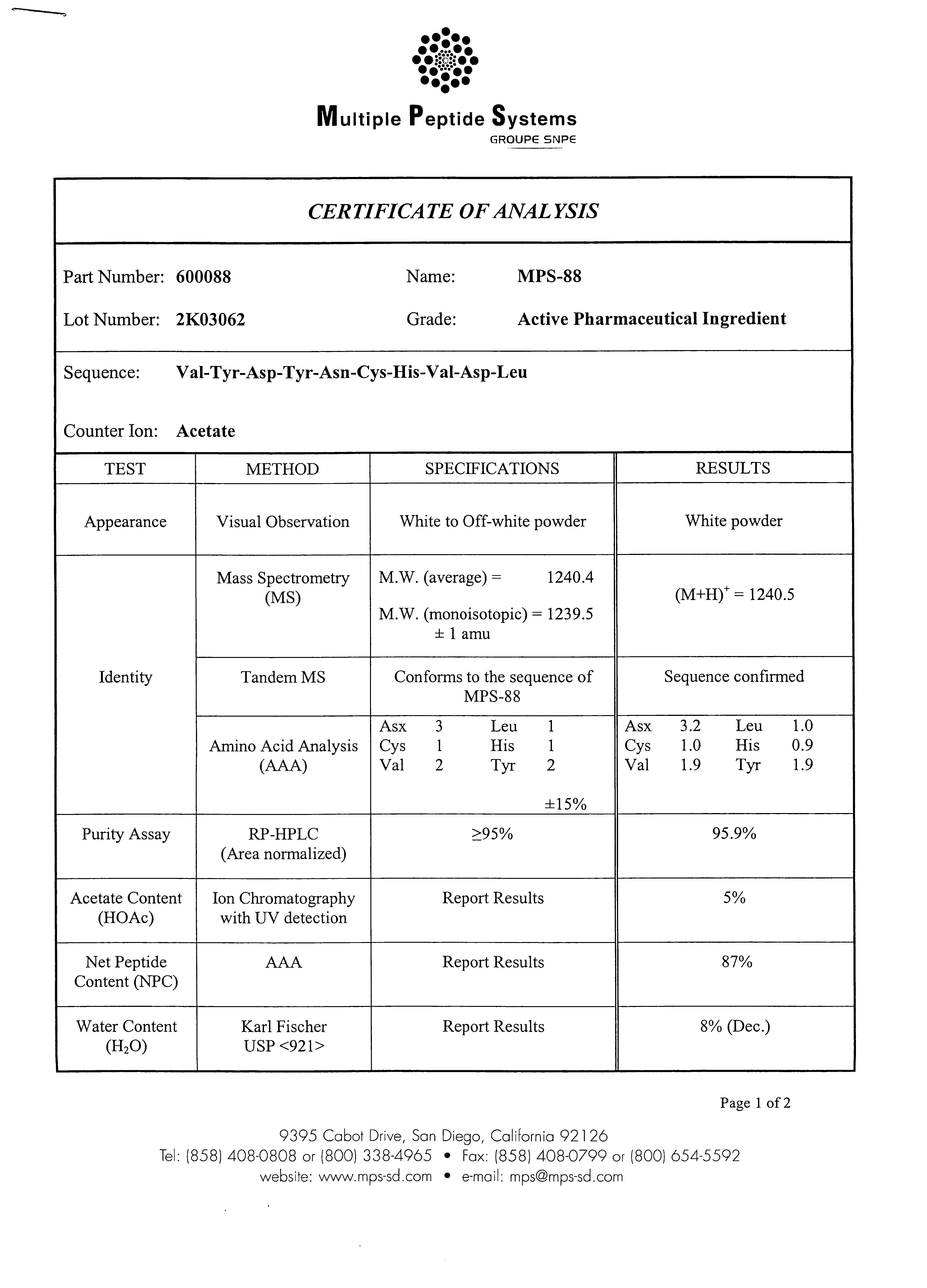


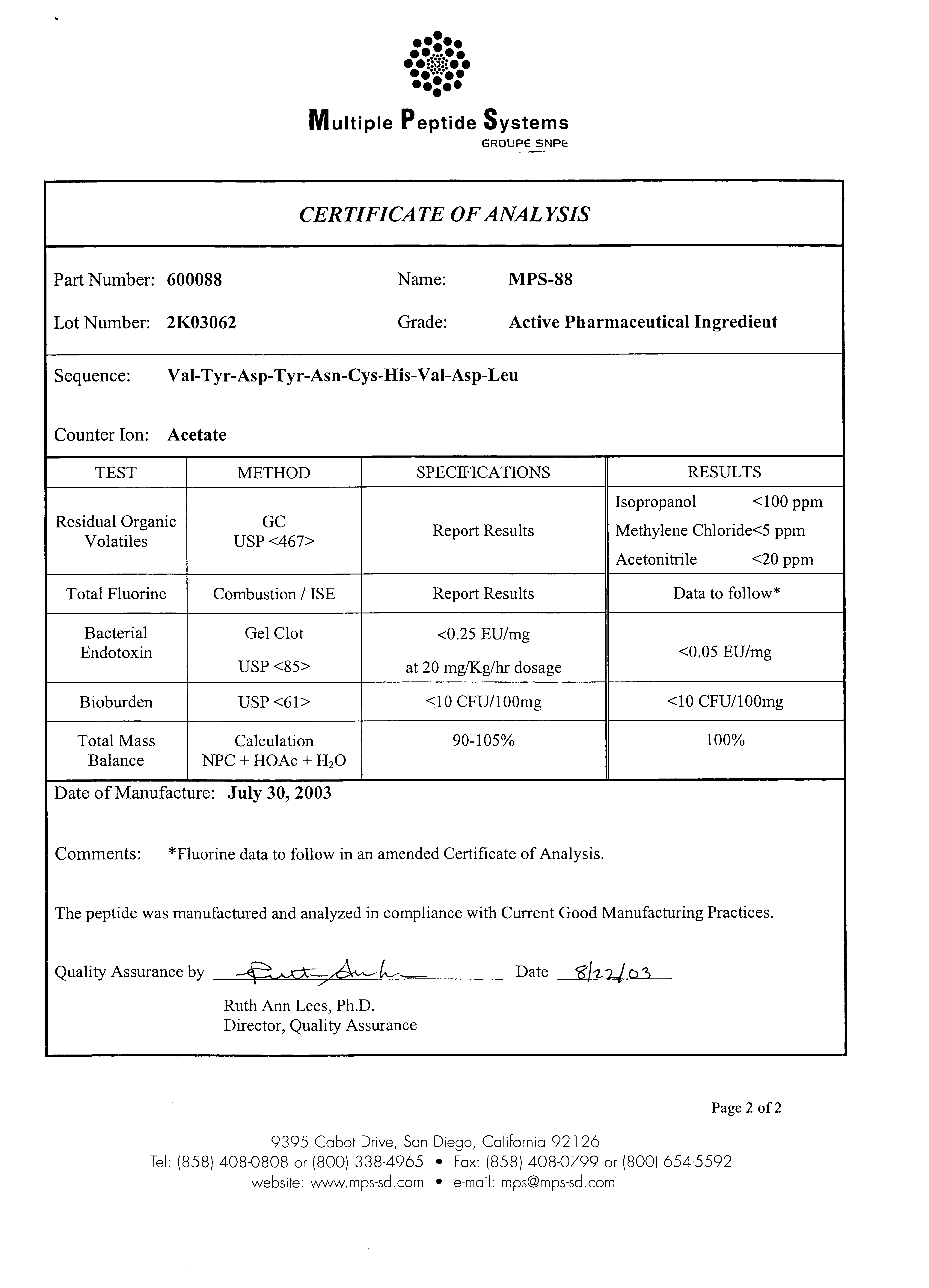


Appendix 2

Weight changes in mice after the administration of peptide vaccine (SART3, MPS-88)

(body weight; g)


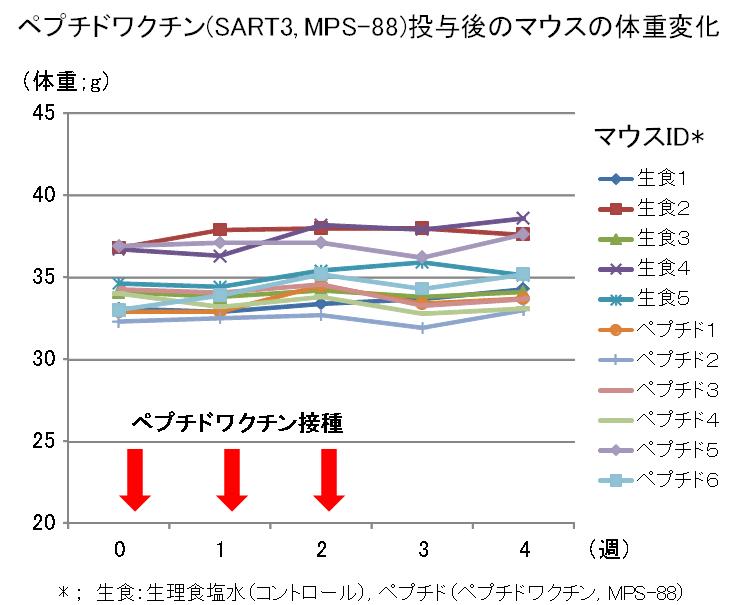


Mouse ID

Saline

Peptide

(Weeks)

*: Saline: saline (control), peptide (peptide vaccine, MPS-88)

In the present study, SART2, SART3, MRP3-derived peptide vaccines are co-administered to mice.
