# Supplementary material for: Cellular Immune Responses for Squamous Cell Carcinoma Antigen Recognized by T Cells 3 in Patients with Hepatocellular Carcinoma
Source: PLoS One. 2017 Jan 23;12(1):e0170291. doi: 10.1371/journal.pone.0170291 (PMC5256867; doi:10.1371/journal.pone.0170291)
Supplement: S2 File — (DOC) [file pone.0170291.s004.doc]

臨床試験「Prp24pヒト相同体RNA結合核蛋白由来ペプチドワクチンを用いた肝細胞がん免疫療法」のための実施計画書

金沢大学

医学倫理審査委員会委員長　　殿

**1.申請事項**

手術療法による根治が不可能な肝細胞がん患者に対する免疫学的抗腫瘍効果を期待して、Prp24pヒト相同体RNA結合核蛋白(SART3)由来ペプチドワクチンを用いた肝細胞がん免疫療法を施行したいので倫理委員会での審査をお願い申しあげます。

**2.ペプチドワクチンの治療概念**

1991年にBoonらによってメラノーマ抗原MAGE遺伝子が同定されて以来、ヒトの免疫系ががんを認識し、排除する働きをもつことが明らかにされてきました。がん排除に働く主な細胞の1つとしてT細胞が挙げられますが、このT細胞はがん細胞の表面に存在する主要組織適合抗原(HLA分子)とがん細胞が産生する蛋白が断片化したペプチドの複合体を認識し、細胞障害活性を示します。

現在までに、多くのがん拒絶抗原とそのアミノ酸配列をもつペプチドが同定され、それらを用いたがん免疫療法の臨床試験が世界中で行われています。これまでの報告では、こうしたペプチドを投与することにより、生体内においてがん細胞を攻撃するT細胞が誘導できること、一部の症例ではがん退縮効果が得られることが明らかにされています（参考文献１）。

**3.臨床試験の目的**

現在、肝細胞がん（肝がん）の治療としては、手術療法、経肝動脈腫瘍塞栓療法（TAE）、ラジオ波焼灼療法（RFA）をはじめとする局所療法、抗がん剤を用いた化学療法が施行されているにもかかわらず、我が国における年間死亡数は、３万人を越えさらに増加傾向にあります。このうち、手術療法において最も高い治療効果が得られていますが、治療の適応は極めて早期のものに限られており、多くの肝がん症例において、手術以外の治療法が選択されているのが現状です。これまで、手術療法により根治不可能な肝細胞がんの治療法として、TAEおよびRFAが施行されてきました。しかし、当科の成績において、TAE治療3年後再発率が88.4 %、RFA治療3年後再発率が75.2%と極めて高い再発率を認め、現状ではこれ以上の生存期間の延長が期待できる治療法が存在しない状態にあります。

そこで、当科では肝がんに対する新たな治療法の開発をめざして、平成13年10月1日～平成15年3月31日の間に手術療法により根治不可能な肝細胞がん患者に対して、臨床試験「樹状細胞を用いた肝細胞がん免疫療法」（金沢大学医学部等医の倫理委員会；受付番号236）を施行しました。この臨床試験では、10名の肝がん患者においてTAE治療前に末梢血から未熟樹状細胞を誘導し、TAE治療の際に経カテーテル的に腫瘍局所に投与を行いました。この臨床試験の結果、未熟樹状細胞が安全に投与されることを確認し、さらにその後、臨床試験「SART-3;109-118ペプチド刺激樹状細胞を用いた肝細胞がん免疫療法」、臨床試験「OK-432刺激樹状細胞を用いた肝細胞がん免疫療法」、臨床試験「ペプチド刺激樹状細胞のブースト投与を行う肝細胞がん免疫療法」を順次施行し、これまでに以下の知見を得ています。

1. OK-432刺激樹状細胞を投与した際の安全性を確認しました。
2. 樹状細胞の投与により肝がんに特異的な腫瘍抗原とそのCTLエピトープに対する免疫反応をTAE単独に比べて効率に誘導できることを確認しました。
3. OK-432刺激樹状細胞を投与することにより、抗腫瘍免疫を誘導でき、同細胞の投与群では、TAE単独治療群に比べて治療後再発を有意に抑制できることを確認しました。
4. 樹上細胞をペプチドで刺激し、このペプチドを結合した樹状細胞のヒトへの投与の安全性を確認しました。

これらの結果からは、肝がん患者において肝がんに対する抗腫瘍免疫を誘導することにより、局所治療後の再発が抑制できる可能性が示されています。また、本臨床試験で使用するペプチド(SART3)は、すでに多施設において臨床試験が行われており、人体に投与することの安全性が確認されています（参考文献２、３）。これまで施行してきた樹状細胞を用いた方法は、治療に際し、患者末梢血より200ccの採血を必要とすること、採取後治療までに1週間の期間を要すること、採取後体外で樹状細胞を培養するため、細菌等の混入が考えられ、特殊な培養環境を要することなどから、より多くの患者に適応を広げることは困難と考えられます。これらの点を克服し、より多くの患者に治療が可能となるように、現在我々は肝がんに特異的な腫瘍抗原エピトープをもつペプチドをヒトの皮下に直接投与することにより、肝がんに特異的な免疫反応を誘導し、局所治療後の再発抑制を目指した治療方法を開発したいと考えています。そこで本臨床試験では、こうしたペプチドを肝がん症例に投与することの安全性と、同方法による抗腫瘍免疫反応の誘導効率について検討することを目的とします。

今回臨床試験に用いるペプチド(Prp24pヒト相同体RNA結合核蛋白由来ペプチド；SART-3；MPS-88)には、これまでに我々が行ってきた肝がん患者の基礎研究において同定された、肝がんに高率に発現している腫瘍抗原由来であり、肝がん患者のリンパ球において高率に認識されるHLA-A24拘束性のCTLエピトープを含んだもの、かつ試験管内でCTLを誘導できるものを選択しました（参考文献４）。また、実際に患者に投与するペプチドには、米国Neo MPS社の品質管理責任のもとに、臨床（GMP）グレードで作製されたもの(MPS-88)（NeoMPS®；ＧＭＰ証明書を添付）（添付資料１）を購入し、使用します。

ペプチドの投与方法は、すでに多くの他施設において施行され、その安全性が確認されているモンタナイドISA-51というアジュバントと混合し、皮下に投与する方法を選択します。なお、当科ではペプチド投与の安全性に関する基礎検討として、体重当り本試験の100倍量のペプチドとアジュバントをマウスに投与して体重の推移や活動性、投与部位の皮膚に変化がないことを確認しております（添付資料２）。

主要評価項目

1. HLA-A24拘束性SART3由来ペプチドワクチン(SART3-109)による有害事象の種類と発現頻度
2. 免疫学的モニタリングによる特異的免疫反応の誘導の観察

副次評価項目

1. 局所治療後再発率の評価
2. 治療終了後の腫瘍マーカーの推移

**4.臨床試験の対象**

手術療法の適応とならない肝細胞がん患者、または同治療を希望しない肝細胞がん患者のうち、TAE^注1)^もしくはRFA^注2)^による治療を行うHLA-A24陽性の患者を対象とします。具体的には、原発性肝がん取扱い規約（第４版、2001年11月）の進行度分類Stage II以上の症例で手術療法の適応がない患者、もしくはStage Iであっても、肝予備能の低下により手術療法が不可能な患者、手術療法を望まない患者であり、以下の基準をすべて満たす12症例を対象とします。このうち先ず3症例に対して1/100(0.03mg)、3症例に対して1/10(0.3mg)のペプチド量を用いた治療を施行します。安全性を確認した後に、残り6症例に3mgのペプチドを投与します。

^注1)^肝動脈塞栓療法；肝細胞がんを栄養する血管に詰め物をすることでがんにいく栄養、酸素を遮断する治療方法。

^注2)^ラジオ波焼灼療法；肝細胞がんに針を刺し、がん局所を熱で治療する方法。

本臨床試験参加基準

●画像所見により、原発性肝細胞がんと確定診断されている。

●Karnofsky Performance Statusが70 % 以上である。

●20歳以上で、インフォームド・コンセントが得られている。

●白血球数 2,000 / mm^3^ 以上である。

●血小板数 50,000 / mm^3^ 以上である。

●ヘモグロビン値 8.5 g/dl 以上である。

●原発性肝がん取扱い規約（第４版）における肝障害度liver damage AまたはBである。

●血清クレアチニン 1.5 mg/dl 以下である。

除外基準：以下の一項目でも該当するものは対象としない。

●心疾患、腎疾患、呼吸器疾患、血液疾患、凝固障害その他の重篤な合併症を有し、担当医師により不適当と判断される。

●HIVに感染している。

●４週間以内に手術、化学療法、放射線療法を受けている。またはこれらの治療から十分に回復していない。

●免疫不全症との診断を受けたことがある。

●副腎皮質ステロイドの投与を必要としている。

●授乳中である。

●妊娠中および妊娠の可能性のある女性。

●同種臓器移植の既往がある。

●追跡経過観察が困難であると予想される。

●プロトコール遵守の不確実性が予想される。

登録者決定までの過程

症例の選定： 担当医師

症例の確認： 責任医師

同意説明： 担当医師

同意書作成： 患者さん、担当医師

治療中の薬剤投与

本臨床試験中に、以下の薬剤投与は禁止とします。

●急性のアレルギー反応治療以外に用いる副腎皮質ステロイドの全身投与。

●免疫抑制剤。

**5.臨床試験の方法**

１）肝細胞がんの局所治療終了後6週以内にペプチド(SART3-109) (MPS-88)を投与します。その後２週間おきに計3回ペプチドを投与します。投与開始前には血液検査にて、患者のHLA class Iのタイピングを行います。

２）ペプチドの具体的な投与方法は、SART3由来ペプチド(SART3-109)（GMPグレード）4mgを生理食塩水1mlに溶解し、5mlの注射器シリンジ（2本）と3方活栓を用いて1mlのモンタナイドISA-51（GMPグレード）と混合し、エマルジョン化させた後、0.5mlずつツベルクリン反応用注射器に分注し、患者皮下（両側腋下）へ、計1.5ml（0.25mlずつ計6回の皮下注）投与します。1/100(0.03mg)、1/10(0.3mg)のペプチド量を投与する症例については生理食塩水1mlに溶解する時点で希釈します。

３）ペプチド投与前、ペプチド最終投与後4週目にそれぞれ末梢血50mlを採取し、ELISPOTアッセイ、テトラマーアッセイにて、ペプチドに特異的な免疫反応が誘導されているかを判定します。この採取量に関しては上記検討方法にてペプチドワクチンに対する生体の反応を詳細に解析するためのリンパ球分離が必要なこと、また、後日予期せぬ副作用出現の際の病態解析に用いるリンパ球を保存しておく目的から、末梢血50mlが必要となります。

また、同時期に生理食塩水にて溶解した10ugのペプチドを用いて遅延型過敏症(DTH)反応を確認します。DTHの判定は、ペプチド溶解液(0.1ml)を患者前腕内側に皮内注射し、48時間後に硬結の直径が4mmを超え、対照とした生理食塩水の反応部位では硬結が観察されないものを陽性とします。

４）治療効果は、肝がん局所療法後3、6、9、12ヵ月の画像診断における腫瘍局所再発率、他部位再発率により判定します。

５）抗腫瘍免疫反応の検討、および予期せぬ副作用出現の際の病態解析の目的でペプチド投与前とペプチド最終投与後4週目にそれぞれ末梢血10mlを採取し、血清分離ののち患者血清を凍結保存します。

６）抗腫瘍免疫反応の検討のうち、T細胞レセプターに関する研究は富山大学医学部医学科免疫学教室に臨床検体を送って行います。

予想される副作用

●発熱

●自己免疫疾患

●肝炎の増悪、肝予備能の低下・肝不全

●細菌感染

●投与部位の皮膚反応；痒み・痛み・発赤・硬結・びらん・潰瘍・痣

●リンパ節腫脹

●白血球増加

●発疹

●アレルギー

●ショック

毒性判定

毒性、有害事象の判定は、NCI Common Toxicity Criteria (CTC) Scale を用いて行います。

試験中止基準

●ヘルシンキ宣言、ICH-GCPガイドライン、米国FDA規則により、被試験者はいついかなる時でも、いかなる理由であっても、以降の医療処置等に影響を与えることなく自由意思で試験を中止する権利を有します。

●重篤な副作用が出現した場合には早急に中止し、適切な治療を実施します。

治療に関する費用

通常の肝細胞がん治療として保険が適用される費用以外は、すべて大学院医学系研究科恒常性制御学研究分野の校費、委任経理金等にて負担します。

健康被害の補償

副作用の出現に十分な注意を払いながら行いますが、本臨床試験により障害、疾病などの健康被害を被った場合はその原因を究明した上で、適切な治療が受けられるよう手配します。しかし、補償は行いません。

データの管理

　患者個人のデータは連結可能な匿名化された状態で入力される。データは臨床試験責任者が厳重に管理し紙媒体については鍵のついた金庫で保管することとする。電子データについてはパスワード設定のされたＰＣで管理する。また本研究において得られたデータは、本研究の目的以外には使用しない。

**6.臨床試験の期間**

本臨床試験の登録期間は、倫理委員会承認後、平成25年3月31日までとします。有害事象観察期間はペプチド投与終了後3ヵ月とし、平成27年3月31日までに解析研究を含めた全臨床試験を終了します。

**7.問い合わせ先**

臨床試験責任者　　　金子周一　　金沢大学医薬保健研究域医学系教授

臨床試験実務担当医　水腰英四郎　金沢大学附属病院消化器内科講師

〒920-8641 石川県金沢市宝町13-1

Tel: 076-265-2235

Fax: 076-234-4250

E-mail: [eishirom@m-kanazawa.jp](mailto:eishirom@m-kanazawa.jp)

添付資料１


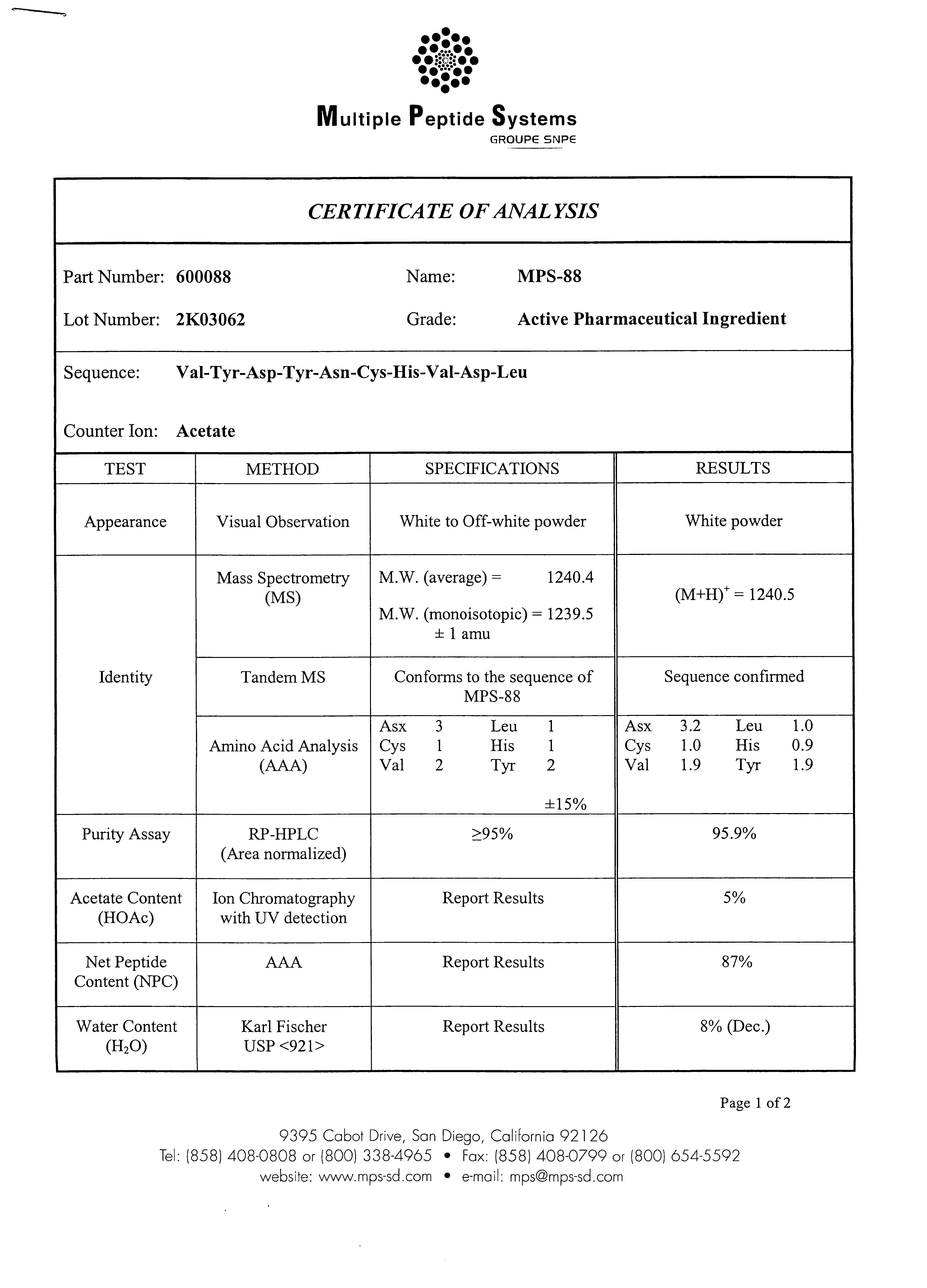


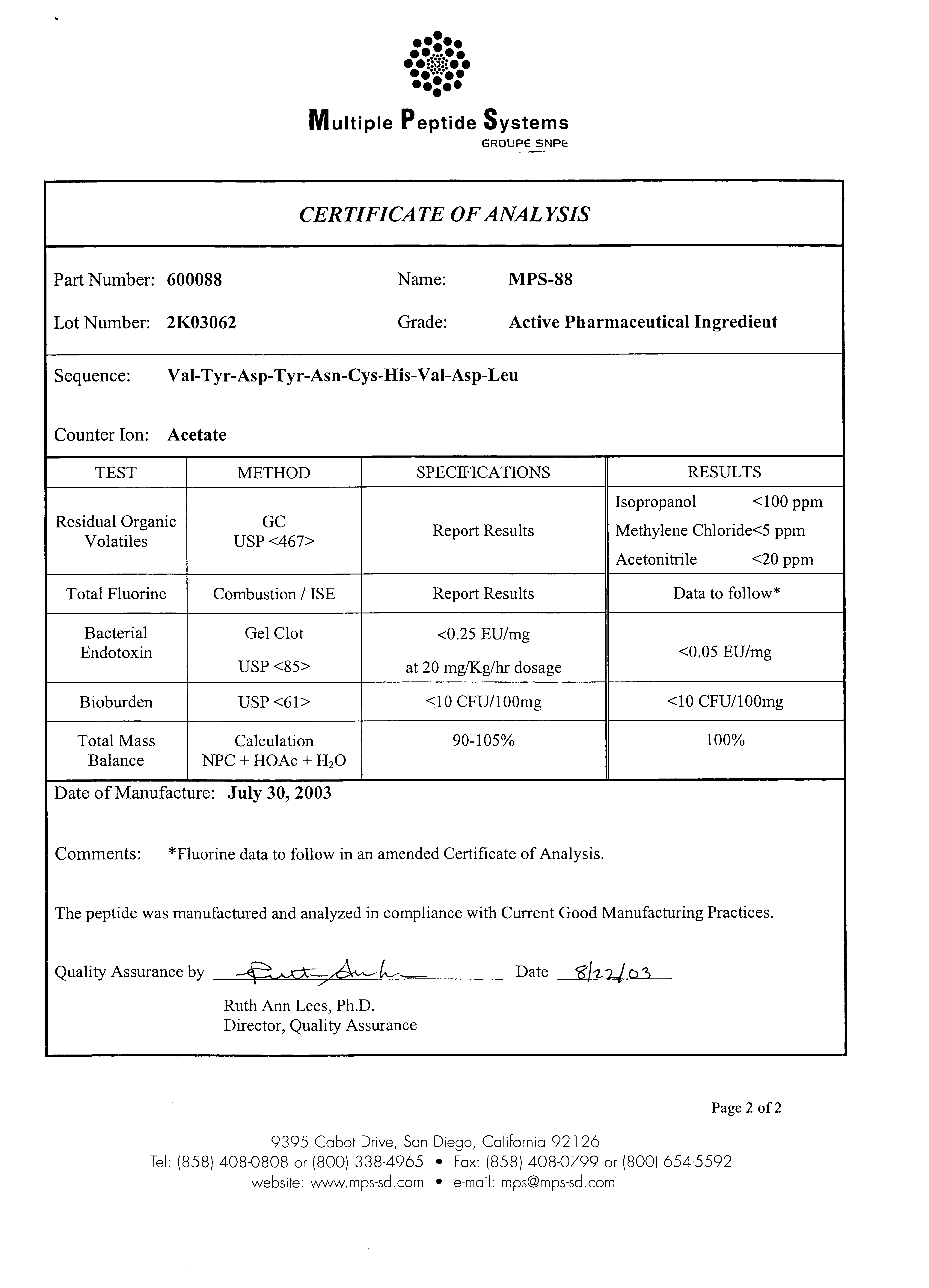


添付資料２


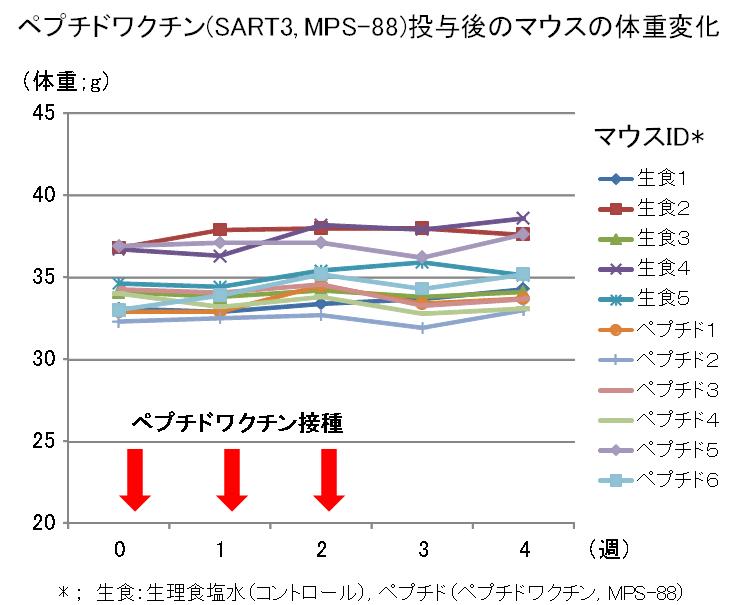


本検討はSART2、SART3、MRP3由来ペプチドワクチンを同時投与したマウスを用いて測定を行っています。
